# Tryptophanhydroxamic Acid-Stabilized Ultrasmall Gold Nanoclusters: Tuning the Selectivity for Metal Ion Sensing

**DOI:** 10.3390/nano14050434

**Published:** 2024-02-27

**Authors:** Gyöngyi Gombár, Ditta Ungor, István Szatmári, Ádám Juhász, Edit Csapó

**Affiliations:** 1 MTA-SZTE Lendület “Momentum” Noble Metal Nanostructures Research Group, University of Szeged, Rerrich B. sqr. 1, H-6720 Szeged, Hungary; gombar.gyongyi@stud.u-szeged.hu (G.G.); ungord@chem.u-szeged.hu (D.U.); 2 Institute of Pharmaceutical Chemistry, University of Szeged, Eötvös u. 6, H-6720 Szeged, Hungary; szatmari.istvan@szte.hu; 3 Stereochemistry Research Group, Eötvös Loránd Research Network, University of Szeged, Eötvös u. 6, H-6720 Szeged, Hungary; 4 Interdisciplinary Excellence Center, Department of Physical Chemistry and Materials Science, University of Szeged, Rerrich B. sqr. 1, H-6720 Szeged, Hungary

**Keywords:** gold nanoclusters, metal ion sensor, hydroxamate derivative of amino acid, fluorescence, combined quenching

## Abstract

Sub-nanometer-sized gold nanoclusters (Au NCs) were prepared via the spontaneous reduction of [AuCl_4_]^−^- ions with a hydroxamate derivative of *L*-tryptophan (Trp) natural amino acid (TrpHA). The prepared TrpHA-Au NCs possess intense blue emission (λ_em_ = 470 nm; λ_ex_ = 380 nm) with a 2.13% absolute quantum yield and 1.47 ns average lifetime. The Trp-stabilized noble metal NCs are excellent metal ion sensors for Fe^3+^, but in this work, we highlighted that the incorporation of the hydroxamate functional group with an excellent metal ion binding capability can tune the selectivity and sensitivity of these NCs, which is a promising way to design novel strategies for the detection of other metal ions as well. Moreover, their simultaneous identification can also be realized. By decreasing the sensitivity of our nano-sensor for Fe^3+^ (limit of detection (LOD) ~11 µM), it was clearly demonstrated that the selectivity for Cu^2+^-ions can be significantly increased (LOD = 3.16 µM) in an acidic (pH = 3–4) condition. The surface-bounded TrpHA molecules can coordinate the Cu^2+^ confirmed by thermodynamic data, which strongly generates the linking of the NCs via the Cu^2+^ ions in acidic pH, and a parallel fluorescence quenching occurs. In the case of Fe^3+^, the degree of quenching strongly depends on the metal ion concentration, and it only occurs when the NCs are not able to bind more Fe^3+^ (~10 µM) on the surface, causing the NCs’ aggregation.

## 1. Introduction

Noble metal nanostructures like nanoparticles (NPs) which possess an average diameter larger than 2 nm as well as unique structure-, composition-, and morphology-dependent plasmonic properties [1] and the ultrasmall nanoclusters (NCs) showing characteristic tunable fluorescence (PL) properties [2,3] play a dominant role in various applications like (bio)catalysis [4], biosensing [5] or diagnostics [6] and therapy [7]. The biomedical utilization of these nanostructured materials requires the design of green fabrication protocols avoiding the use of extra additives (molecules helping the reduction of precursor metal ions or stabilizing ligands) and toxic substances (e.g., organic solvent, harsh reducing agents, etc.). Based on these requirements, in the last 10 years, several production processes have emerged, where only one smaller or larger molecule like proteins [8,9], peptides [10] and simple amino acids [11,12,13] are used for the reduction of metal ions (e.g., [AuCl_4_]^−^, Ag^+^ or Cu^2+^), which in addition play a dominant role in the stabilization and functionalization of the metallic surface as well. In the case of amino acids, several articles can be found for tryptophan (Trp) [12,14,15,16,17,18,19,20], tyrosine (Tyr) [21,22], histidine (His) [12,13,23], arginine (Arg) [24], or amino acids containing S donors like cysteine (Cys) or methionine (Met) [11,13]. Among these amino acids for Trp, it was confirmed that Au- [12,15,16,18,20], Ag- [14,19], and Cu-based [18] NCs can also be produced via a direct reduction of the metal ions with Trp without the application of any other agents. By comparing the experimental conditions optimized in these publications, it can be stated that predominantly the use of ligand excess (depending on the metal ions, these are in the range of a 2:1–20:0 molar ratio, 100–120 °C temperature, and basic conditions (pH = 11–12)) can be generally used to produce blue-emitting Trp-coated NCs. Changing these experimental conditions, in our previous work [12], we confirmed that the synthesis of Trp-Au NCs can also be carried out in an extremely acidic medium (pH = 1–2) and thus does not require boiling; the reduction of metal ions takes place at 37 °C within 24 h. This latter reaction is based on the autopolymerization of indole ring-containing compounds in highly acidic aqueous medium [25], and thus the other functional groups like carboxylate and amino do not suffer structural changes. Besides amino acids, their derivatives [15,26] could also be potential bioligands to produce NCs using the template-assisted green fabrication method, where the bioligand acts as a reducing and stabilizing molecule as well. Among these molecules, the hydroxamic acid derivatives of amino acids have excellent metal ion binding properties, and thus the formation of hydroxamic acid group(s) on the surface of NCs allows for the formation of a potential metal ion sensor. Only one article is available in the literature for the histidinehydroxamic acid-directed synthesis of Au NCs [26], which was published by our group. Here, we confirmed that the presence of the hydroxamate moiety highly increases the sensitivity for Cu^2+^. Continuing our work, in this paper, we demonstrate a new preparation method for the fabrication of gold-based NCs using TrpHA as a reducing, stabilizing and functionalizing agent, which has not been reported in the literature. Using the above-mentioned method designed for the synthesis of Trp-Au NCs in an extreme acidic medium [12], our aim was to efficiently create Au NCs such that the hydroxamate functional group with an excellent metal ion binding property is available on the surface without structural change. In addition to the optimization of the synthesis and the detailed structural characterization of the NCs, their potential sensing applications are also presented, where we highlight the identification possibility of Cu^2+^ in the presence of Fe^3+^ to a given concentration, and the quenching mechanisms are also interpreted.

## 2. Materials and Methods

### 2.1. Materials

Tryptophanhydroxamic acid (C_11_H_13_N_3_O_2_, TrpHA) was synthesized based on the protocol presented in Chapter 2.2. *L*-Tryptophan (C_11_H_12_N_2_O_2_, ≥99%) and hydrogen tetrachloroaurate (III) monohydrate (HAuCl_4_ × H_2_O, 99.9% (metal basis)) were purchased from Sigma-Aldrich (Budapest, Hungary). Metallic salts used for sensing tests, such as rubidium chloride (RbCl_2_; 99.8%), copper(II) chloride (CuCl_2_, 97%), yttrium(III) chloride (YCl_3_; 99.99%), lanthanum chloride hydrate (LaCl_3_ × H_2_O; 99.9%), magnesium chloride (MgCl_2_; ≥98%), iron(III) chloride hexahydrate (FeCl_3_ × 6 H_2_O, 99.9%), cerium chloride heptahydrate (CeCl_3_ × 7 H_2_O; 99.9%), cobalt(II) chloride hexahydrate (CoCl_2_ × 6 H_2_O; 98%), rhodium chloride (RhCl_3_; 98%), manganese(II) chloride tetrahydrate (MgCl_2_ × 4 H_2_O; 98%), nickel(II) chloride (NiCl_2_, >98%), zinc(II) chloride (ZnCl_2_; 99.9%), calcium chloride dihydrate (CaCl_2_ × 2 H_2_O; 97%), cadmium nitrate tetrahydrate (Cd(NO_3_)_3_ × 4 H_2_O; 98%), cesium chloride (CsCl; 99%) and potassium chloride (KCl; >99%) were also obtained from Sigma-Aldrich. Sodium hydroxide (NaOH, 99%), sodium chloride (NaCl, 99.98%) and hydrochloric acid (HCl, 37%) were purchased from Molar (Halásztelek, Hungary). 

Since all the compounds were analytical grade, no additional purification was carried out. Milli-Q ultrapure water (Merck Millipore, Merck Kft., Budapest, Hungary) (18.2 MΩ·cm at 25 °C) was used to prepare the stock solutions freshly.

### 2.2. Synthesis of TrpHA

The TrpHA ligand was synthesized according to previously published preparation protocols, as Figure S1 presents [27,28,29]. *L*-tryptophan methyl ester hydrochloride was used as a starting ligand, and different optimization steps were carried out. As the first step, in a flask with a round bottom, 1.0 g (3.93 mmol) of *L*-tryptophan methyl ester hydrochloride was added and dissolved in 8 mL of water. After that, 20 mL of hydroxylamine solution (50 wt % in H_2_O) was also added to this aqueous solution, and the reaction mixture was stirred at 25 °C. Following the attainment of maximum conversion, which was reached after a 24 h reaction period and the thin-layer chromatography (TLC) showed no more starting material, the mixture was cooled using an ice bath, and the crystals that were created were filtered and cleaned with cold water (2 × 5 mL). Yield: 474 mg (55%); M.p.: 183–185 °C; ^1^H-NMR (DMSO-d6, Figure S2) δ: 2.68–2.76 (1H, m); 2.95–3.04 (1H, m); 3.34–3.39 (1H, m); 6.95 (1H, t, *J* = 7.3 Hz); 7.05 (1H, t, *J* = 7.4 Hz); 7.12 (1H, s); 7.32 (1H, d, *J* = 7.8 Hz); 7.55 (1H, d, *J* = 7.9 Hz); 10.80 (1H, s); MS (Figure S3): [M+H]^+^ *m*/*z* = 219.99.

### 2.3. Synthesis of TrpHA-Directed Au NCs

Initially, 0.789 mL of TrpHA aqueous solution at a concentration of 0.01268 M was added to 3.711 mL of Milli-Q ultrapure water. After 2 min stirring, the pH of the solution was adjusted to pH = 2.0 with 0.1 M hydrochloric acid, and 0.500 mL of 0.01 M HAuCl_4_ solution was added. After mixing the components, the sample was thermostated at 80 °C ± 1 °C for 48 h. According to the quantity of each component, the TrpHA:[AuCl_4_]^−^/2:1 molar ratio was applied, while the final amount of the gold was 1.0 mM. At the end of the synthesis time, the initial intense yellow color of the [AuCl_4_]^−^- ions gradually faded during the reduction process and finally turned to pale brown, indicating the formation of the final fluorescent product. For the last step, some aggregates were centrifuged for 30 min using 13,000 rpm, followed by the elimination of the excess alkali and the residual unreduced precursor metal salt from the sample via dialysis for 120 min using a cellulose tube with 1 kDa cut-off (Pur-A-Lyzer TM Mega 1000, Sigma-Aldrich Ltd. (Budapest, Hungary)). After dialysis, only ~5% of the gold content was lost, resulting in 0.95 mM gold concentration. This is confirmed by experiments presented in 3.1 Chapter (Figure S5). The purified aqueous dispersion was stored in a normal fridge (+4 °C). Moreover, one part of the purified aqueous dispersion containing the TrpHA-Au NCs were freeze-dried by with a Christ Alpha 1–2 LD plus device (DONAU LAB Budapest, Hungary) for FT-IR measurements. The freezing of the aqueous dispersion containing the clusters was performed with the use of liquid nitrogen. Under 4 mbar pressure, the solvent was evaporated (~20 h), and the dried lyophilized powder was stored in a freezer (−20 °C) until use.

### 2.4. Instruments for Characterization

Fluorescence spectra of the NCs-containing aqueous dispersion after dialysis (without dilution) were recorded on the ABL&E JASCO FP-8500 spectrofluorometer (ABL&E JASCO, Budapest, Hungary) using a standard quartz cuvette with 1 cm path length. Measurements were performed by using 380 nm as an excitation wavelength, 2.5 input and 2.5 nm output bandwidths, 1 nm resolution, and a scan speed of 200 nm/min. For the determination of the absolute quantum yield, the above-mentioned fluorometer was applied which contains an ABL&JASCO ILF-835 integrating sphere (ABL&E JASCO, Budapest, Hungary). In the case of the detector calibration, an ABL-JASCO ESC-842 WI calibrated lamp (ABL&E JASCO, Budapest, Hungary) was used. The time-correlated single photon counting (TCSPC) technique was used for the determination of fluorescence lifetime. For this purpose, a Horiba DeltaFlex device containing a DeltaDiode pulsed laser (λ_em_ = 371 nm) as an excitation light source was used (HORIBA Scientific, Kyoto, Japan), while the quartz cuvette had 1 cm optical length. The instrument response function (IRF) was registered by SiO_2_ (d = 50 nm, Horiba) colloid particles. The recorded decay curves were analysed by the Horiba EZTime 2.0 program. The UV–Vis spectra of the original dispersions were registered on the ABL&E-JASCO V-770 spectrophotometer (ABL&E JASCO, Budapest, Hungary) using 1 cm optical path length in the wavelength range of 300–600 nm. Fourier-transformed infrared spectroscopy (FT-IR) was applied to determine the possible coordination between the gold metal cores and the stabilizing of TrpHA on the metallic surface, based on measurements with a JASCO FT/IR-4700 instrument (ABL&E JASCO, Budapest, Hungary) equipped with an ATR PRO ONE Oneflexion accessory. FT-IR spectra were registered in 500–4000 cm^−1^ wavenumber range with 128 interferograms at 1 cm^−1^ resolution on lyophilized powders. The corresponding spectrum of the TrpHA was also registered in powder form using the same concentration and pH. The ζ-potential values of the diluted dispersions (four times diluted) were determined with a Malvern Zetasizer NanoZS ZEN 4003 apparatus (Malvern Panalytical, Cambridge, UK), which is equipped with a He-Ne laser (λ = 633 nm), at room temperature.

### 2.5. Interactions between TrpHA-Au NCs with Mono-, Di- and Trivalent Metal Ions 

In all cases, individual aqueous samples with 2.0 mL total volume containing the NCs and the metal ions at constant concentrations were used for sensor studies. Namely, the following procedures were applied: To 250 µL of TrpHA-Au NCs, 1.0 mL of sodium chloride solution was added to ensure a constant ionic strength (c_NaCl_ = 1.0 M in the sample). To study the effect of the metal ions used, (Ca(II), Ce(IV), Cd(II), Fe(III), Co(II), Cu(II), Cs(I), K(I), La(III), Mg(II), Ni(II), Rb(I), Rh(III), Yt(III), Zn(II), Al(III)), 200 µL of metal ion solution was separately added to the above-mentioned sample, where the metal ion content was 1.0 mM. To investigate the quenching effect of Cu(II) and Fe(III), detailed fluorescence quenching studies were also performed in the concentration range of 1 nM–100 mM. The emission spectra of Au NCs were registered at 25 °C, both in the presence and in the absence of the metal ions of interest. To avoid misinterpretation of the selectivity, the correction of the recorded emission spectra was performed according to Equation (1):

(1) $$I_{corr}=I_{m}\times 10^{(A_{EX}+A_{EM})/2},$$

where I_corr_ is the corrected PL intensity, and I_m_ is the PL intensity measured at 470 nm (TrpHA-Au NCs). A_EX_ and A_EM_ are the absorbance measured at the excitation wavelength (λ_ex_ = 380 nm) and the absorbance measured at the emission wavelength (λ_em_ = 470 nm), respectively. For the evaluation of the data, I_corr_ values are expressed as I. Detailed quenching studies were conducted at several temperatures (T = 10 °C; 20 °C; 25 °C; 30 °C; 35 °C) as well to give more information on the main thermodynamic parameters of the interaction between the NCs and the Cu^2+^ ions. The procedure for evaluating the thermodynamic data is described in the Results and Discussion section. To characterize the interaction between the TrpHA-AuNCs and Fe(III)ions, isothermal titration calorimetry (ITC) measurements were performed via a Malvern Panalytical (Kassel, Germany) MicroCal VP-ITC instrument, while the raw data were evaluated with the supplied Microcal add-on for Origin 7.0 (Additive GmbH, Friedrichsdorf, Germany). The Fe^3+^ solution (c_iron(III)_ = 2.0 mM) was titrated into the sample cell filled with 1.43 mL of the TrpHA-Au NCs aqueous dispersion (c_Au content_ = 1.0 mM). Titration was accomplished in 38 consecutive dosing steps with a volume of 7 μL Fe^3+^ solution and with a constant stirring rate of 240 rpm and a time of 900 s between each injection. The background heat of the dilution was also determined, at the same conditions of the respective experiment, via the titration of Fe^3+^-containing solution with pure Milli-Q water and subtracted from the adsorption heat.

## 3. Results and Discussion

### 3.1. Optimization of the Preparation Protocol

**Figure 1:**
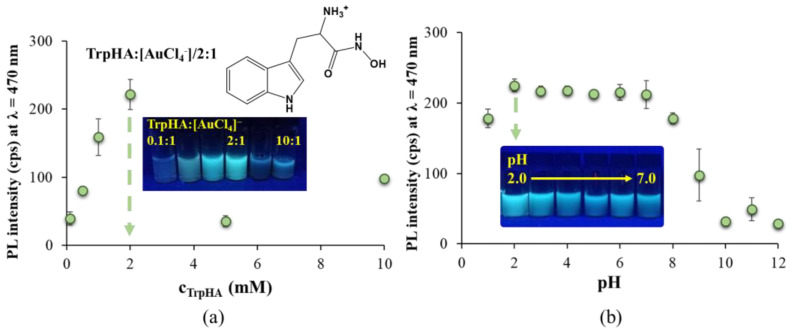
(**a**) The maximum emission intensity values of the fluorescent products at λ_em_ = 470 nm as a function of c_TrpHA_ after 24 h (c_Au_ = 0.95 mM, pH = 7.0, λ_ex_ = 380 nm) with the fully protonated structural formula of the TrpHA and a photo of the samples using different TrpHA/metal ion molar ratios. (**b**) Fluorescence intensities detected at λ_em_ = 470 nm as a function of the pH (c_Au_ = 0.95 mM, TrpHA:[AuCl4]^−^/2:1, λ_ex_ = 380 nm) with the photos of the samples between pH = 2 and 7.

To find the optimal experimental conditions, like the ratio of metal ion to ligand, the initial concentration of the precursor metal ion, temperature, pH and reaction time, several individual experiments were carried out. As an initial step of our work, we investigated the effect of TrpHA: [AuCl_4_]^−^ molar ratios on the appearance of the fluorescent product(s). The syntheses were carried out in the range of 0.1:1.0–10:1 ligand to metal ion ratios. The emission spectra are summarized in Figure S4, while the maximum emission intensities of the final product(s) at λ_em_ = 470 nm as a function of the applied c_TrpHA_ are presented in Figure 1a.

**Figure 2:**
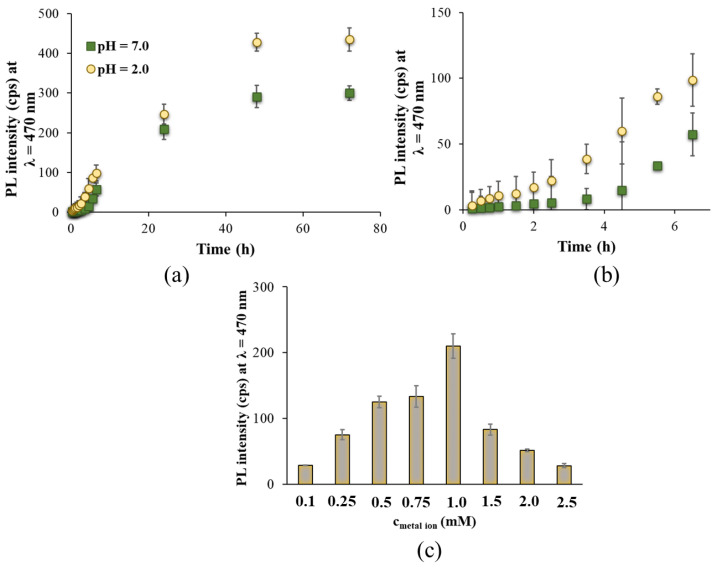
PL intensity values of the sub-nanometer-sized products at different time points ((**a**): 0–80 h, (**b**): 0–6 h) (TrpHA:[AuCl_4_]^−^/2:1, λ_em_ = 470 nm, λ_ex_ = 380 nm, c_Au_ = 0.95 mM, pH = 2.0 (●) and 7.0 (■). (**c**) Effect of metal ion concentration on the PL intensities of the NCs-containing dispersion (TrpHA:[AuCl_4_]^−^/2:1, c_Au_ = 0.95 mM, pH = 7.0, λ_ex_ = 380 nm, λ_em_ = 470 nm).

For all studied ligand to metal ion ratios, the same characteristic emission band is observed, as Figure S4 presents; only the intensities are varied, indicating the formation of the same product(s) with a different yield. We observed that the application of a TrpHA: [AuCl_4_]^−^/2:1 molar ratio results in the formation of stable Au nanostructures with the most intense bluish-green emission at an excitation wavelength of λ_ex_ = 380 nm at pH ~7.0. In the case of a smaller ligand excess (e.g., TrpHA: [AuCl_4_]^−^/1:1), there are not enough ligands in the solution to reduce all the metal ions which results in NCs with less yield, and thus the characteristic emission intensity does not reach the maximum level. If we use higher amounts of the ligand (e.g., TrpHA: [AuCl_4_]^−^/5:1), the presence of large amounts of the ligand may sterically inhibit nucleation, which also results in a decrease in fluorescence intensity. The pH has also important effect on the appearance of fluorescent NCs, so the syntheses were carried out at several pH as well. The emission intensities of the systems are presented in Figure 1b. We observed that the acidic pH range is more favorable than the basic condition (nearly the same PL intensity can be detected in the pH = 2–7 range). Based on similar values, pH = 2.0 was chosen for further studies. Comparing the effect of the ligand to metal ion molar ratio and the pH on the formation of TrpHA-Au NCs, similar results can be obtained than it was found for pure Trp-containing systems. Namely, for Trp-stabilized blue-emitting NCs (λ_em_ = 497 nm at Trp:[AuCl_4_]^−^/1:1; λ_em_ = 486 nm at Trp:[AuCl_4_]^−^/5:1), the acidic condition (pH ~1–2) as well as a lower ligand excess (Trp:[AuCl_4_]^−^/1:1—5:1) were optimal [12]. When investigating the effect of temperature (Figure not presented), we found that increasing the temperature from 25 °C to 80 °C results in higher PL intensities; the maximum is reached at 80 °C, while negligible PL can be observed at lower temperatures. Boiling the sample causes the presence of larger NPs and aggregates, so 80 °C was accepted as appropriate. To find the ideal synthesis time, the emission spectra of the reaction mixture were recorded at many points in time. These experiments were carried out at pH = 7.0 and pH = 2.0 as well (Figure 2a,b) to further confirm the determinative role of the pH. The fluorescence intensities increase nearly linearly to 24 h, but the maximum value is reached after 48 h. It can be noted that there is a kinetically favorable reaction pathway for the formation of TrpHA-Au NCs because of the well-known autopolymerization of indole-containing compounds in a highly acidic environment [25], which is also the driving force for cluster formation in the Trp-Au NCs system. As the last step in the preparation, the effect of the metal ion concentration was also studied in the range 0.1–2.5 mM. Based on the recorded PL spectra (Figure 2c), the highest emission was observed at c_metal_ = 1.0 mM. The application of a higher metal content led to a decrease in PL intensity, and smaller aggregates also appeared in the sample. After synthesis, the reaction mixture was purified via centrifugation at 13,000 rpm for 30 min, after 10 min of sonication, and via dialysis for 120 min to remove the excess alkali and the residual unreduced precursor metal salt from the sample. Before centrifugation via 10 min of sonication, a small amount of NCs attached to the surface of the glass was redispersed into the dispersion. This is confirmed by the emission spectra of the NCs’ dispersion after the 48 h synthesis time because the emission intensity can be increased by ca. 8% (Figure S5). At the end of the 120 min dialysis, the intensity of the dispersion decreased by only about 5% so that the degree of the diffusion of the NCs through the membrane was low, which was considered when calculating the NCs’ concentration.

### 3.2. Structural and Optical Features of the Fluorescent NCs

**Figure 3:**
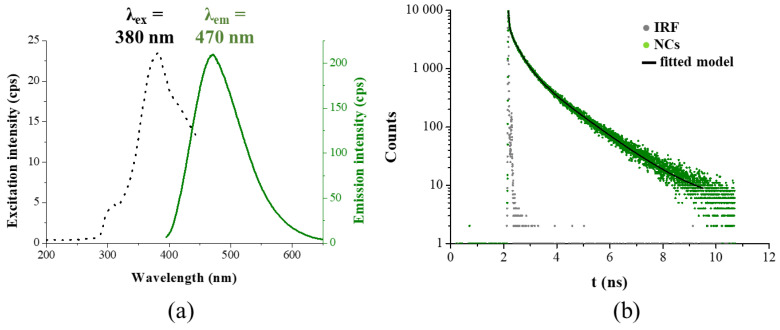
(**a**) Representative excitation (black dots) and emission (green continuous line) spectra of TrpHA-Au NCs (c_Au_ = 0.95 mM). (**b**) PL decay curve (green dots) of the TrpHA-Au NCs, the IRF (gray dots) and the fitted model (black line) (λ_ex_ = 371 nm).

For optical characteristics of the purified TrpHA-Au NCs, the excitation and emission spectra (Figure 3a), the PL lifetime and absolute quantum yield values (Figure 3b) are determined. For the emission spectrum, only one characteristic band at 470 nm can be identified and the presence of other band(s) at lower wavelength is not observed, which means that the oxidation of Trp amino acid to indole-3-acetic acid supported by Z. S. Kardar [18] is probably blocked by the presence of the hydroxamate functional group. The appropriate absorbance spectra of the pure ligand and the TrpHA-Au NCs are presented in Figure S6. The determined absolute quantum yield of the freshly prepared NCs is 2.13%, which is in good agreement with previously determined values for Trp-stabilized Au [20] and Cu [18] NCs. The calculated average PL lifetime is 1.47 ± 0.18 ns, which originates from three components thanks to the different photophysical processes. The main components are the following: τ_1_ = 12.85 ns with 47.8%, τ_2_ = 3.57 ns with 37.4% and τ_3_ = 0.27 ns with 14.8%. In general, it can be said that the shortest τ_3_ value belongs to the few atomic metallic seeds, while the longer τ_1_ and τ_2_ represents the surface ligand-dependent metal–ligand charge transfers. Based on the well-known Jellium model [30], which provides a quantitative relationship between the electronic structure of the cores in the metal NCs and the number of metal atoms (E_Ex_ = E_Fermi_/N^1/3^, where E_Ex_ is the excitation energy, E_Fermi_ is the Fermi energy of bulk gold (5.52 eV) and N is the number of Au atoms), one cluster core probably contain ~five Au atoms.

Infrared spectroscopy is a widely used analytical method to identify the forming bond(s) between the donor groups of ligand molecule and the metal surface or to highlight the change in the structure of dominant functional groups before and after the oxidation of the amino acid during the NCs’ synthesis. Figure S7 shows the infrared spectra of the pure TrpHA and the TrpHA-Au NCs at the same pH using lyophilized powders. TrpHA is a nitrogen-containing heterocyclic compound with a pyrrole ring fused to a benzene ring and a hydroxamic acid and amino groups. Based on the spectra, we determined that the symmetric stretching vibration of the -NH group of the indole ring is shifted from 3397 cm^−1^ to 3382 cm^−1^ due to an interaction with the aurate ions, which suggest that this group has role in the oxidation of the Trp as well as in the metal ion binding. The shift of the characteristic bands (e.g., ν_C=O_ δ_N-H_, δ_NOH_, ν_C-N_)—presented with a continuous line in the IR spectra—is not detected. S. Li et al. synthesized Trp-coated Ag NCs. They suggested that Ag^+^ is reduced to Ag NCs by the N–H in the Trp, and the N–H was oxidized to –NO_2_, which can be confirmed by FT-IR measurements [15]. In our case the dominant presence of –NO_2_ cannot be identified by infrared studies. Moreover, the oxidation of the hydroxamate acid moiety results in the appearance of a nitroso group-containing compound, which, similar to –NO_2_, cannot be detected by IR. Most probably in our case, the NCs’s formation is based on a polymerization reaction, which is highly preferred in extreme acidic conditions for molecules with an indole ring [25], but many previous studies have demonstrated the presence of a relatively strong binding affinity between primary amine groups in organic ligands and the surface of Au clusters, so the binding of amino-N can also be assumed. Thus, hydroxamate moieties can bind metal ions.

**Figure 4:**
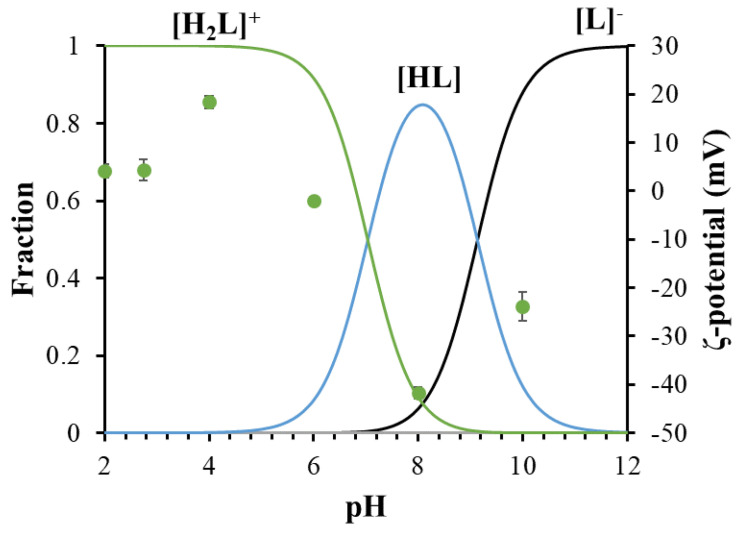
Concentration distribution curves of TrpHA as a function of pH (c_TrpHA_ = 2.0 mM, continuous lines) with the ζ-potential values (marked with green dots) of the TrpHA-Au NCs as a function of pH (c_Au_ = 1.0 mM, I = 1.0 M NaCl).

Because of the ultrasmall size of the NCs (probably containing ~five atoms), neither high-resolution transmission electron microscopy nor the dynamic light scattering (DLS) technique results in valuable data on individual (primer) NCs size and size distribution. The best HRTEM images are presented in Figure S8. However, a size ~0.97 ± 0.41 nm was measured by DLS, which may belong to the presence of individual clusters (sub-nanometer-sized domain containing two–three cluster cores), but since this value is close to the detection limit of the instrument, it can only be accepted hypothetically. Slightly larger domains (aggregates) formed by the interconnection of a few tens of nanoclusters can be clearly identified in the sample by HRTEM (Figure S8) and DLS, so based on the appearance of a surface, zeta potential values can be determined between pH = 2 and 10. Based on these measurements, the binding of TrpHA on the metallic surface was clearly confirmed because the change in the ζ-potential values as a function of pH perfectly follows the concentration distribution curve of the TrpHA molecule [31,32]. Namely, well-defined positive values (e.g., ζ-potential = +20.15 ± 1.5 mV at pH ~4.0) can be measured below pH ~6, where the TrpHA is in a fully protonated form (H_2_L^+^) (Figure 4). Parallel with the proton dissociation (from ammonium to the amino group), the ligand becomes neutral, and the ζ-potential changes its sign, indicating the presence of the ligand on the metallic surface. To study the salt tolerance of the purified NCs, the effect of NaCl concentration on the stability of the NCs was studied in the range of 0.25–2.5 M. Dominant changes regarding the emission intensity of the NCs were not detected below ~1 M of c_NaCl_, but a higher salt content causes a measurable decrease in PL intensity (ca. 30%), so for sensor investigations, c_NaCl_ = 1.0 M was used. The samples do not show any sensitivity under ambient light, but rather the effect of temperature is the determining factor for storage. Namely, the purified aqueous dispersion can be freeze-dried, and the lyophilized powders are stored in −20 °C for months. On the other hand, the purified aqueous dispersion is stored in a normal fridge (+4 °C), and after 2–3 weeks, no structural change or aggregation can be observed. At room temperature within 24 h, aggregation does not occur, but after 48 h, mild flocculation can be detected.

### 3.3. Interaction of the NCs with Different Metal Ions

**Figure 5:**
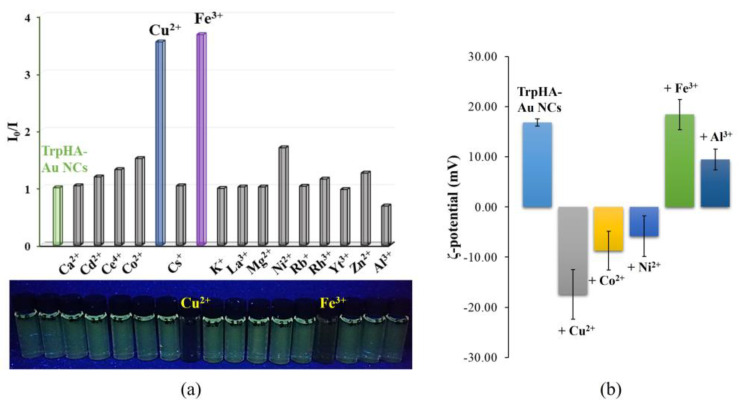
(**a**) Corrected relative PL and (**b**) ζ-potentials of the of TrpHA-Au NCs after addition of several metal ions (c_metal ions_ = 1.0 mM, c_Au_ = 0.25 mM, c_NaCl_ = 1 M, T = 25 °C, λ_ex_ = 380 nm, λ_em_ = 470 nm) with a photo of the individual samples under UV lamp.

Biologically important metal ions as well as generally tested metal ions were used in our experiments; the studied metal ions were listed in Section 2.5. It is well known that metal ions with a paramagnetic feature like Fe^3+^, Cu^2+^, Co^2+^, Ni^2+^, etc., cause PL quenching with great frequency, while for diamagnetic metals like Al^3+^ or Zn^2+^, PL enhancement is quite common. The selectivity and the sensitivity are greatly controlled by the quality of the bioligand, which stabilizes the metallic core. For the application of TrpHA-Au NCs as a potential optical sensor, the PL signal of the NCs was recorded in the absence as well as in the presence of several metal ions. The relative fluorescence values (I_0_/I) are presented in Figure 5. As it was published in the literature [14,18,20], for Trp-Au NCs, measurable fluorescence quenching could be observed only in the case of Fe^3+^. If we modify the carboxylate group to the hydroxamate for the TrpHA-containing system, dominant quenching was caused by Cu^2+^ and Fe^3+^ ions (I_0_/I ~3.6–3.8) as well. For Ni^2+^ and Co^2+^, only a slight degree of quenching (I_0_/I ~1.3–1.5) could be detected. Based on the results, the limit of detection (LOD) was utilized to determine, regarding Cu^2+^ and Fe^3+^, which caused the dominant quenching. For this reason, the PL quenching experiments were conducted at various concentrations, where the amount of the metal ions was varied in the 1.0 nM–100.0 mM concentration range.

**Figure 6:**
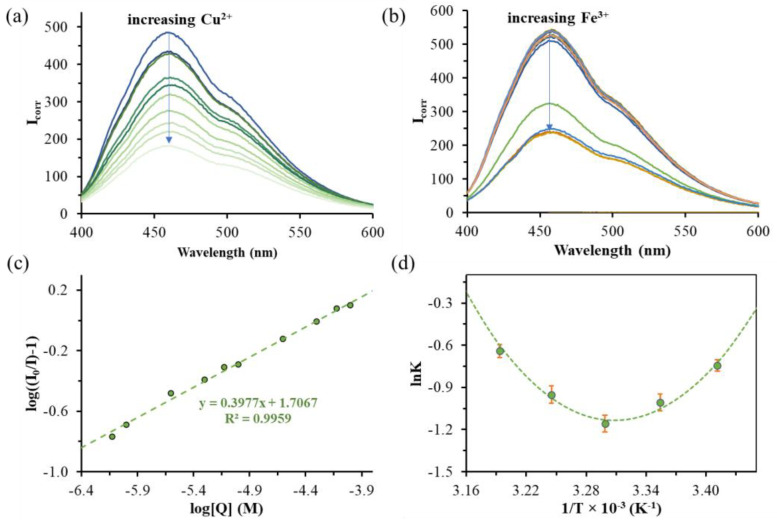
Emission spectra of TrpHA-Au NCs in the presence of increasing concentrations of Cu^2+^ (**a**) and Fe^3+^ (**b**) ions (c_Au_ = 0.25 mM, λ_ex_ = 380 nm, t = 25 °C). (**c**) Modified Stern–Volmer representation of PL quenching data of TrpHA-Au NCs (c_Au_ = 1.0 mM) in the presence of increasing concentrations of Cu^2+^ at 25 °C. (**d**) Van ‘t Hoff representation of ln K_b_ vs. 1/T data (●) for Cu^2+^ with TrpHA-Au NCs interaction (dashed green curve represents the result of the nonlinear parameter estimation).

In the case of Cu^2+^, a nearly continuous PL intensity decrease can be observed (Figure 6a), while for Fe^3+^, an interesting, staggered fluorescence quenching is detected (Figure 6b), which is strongly based on the Fe^3+^ concentration. Based on these experiments, the LOD can be identified for only Cu^2+^, which is 3.16 µM [33]. As Figure 6c represents, a linear relationship can be obtained in the concentration range of ~1.5–90 µM. With further increases in the concentration, we obtain a curve tending toward saturation, which indicates a combined (static + dynamic) quenching mechanism. Parallel with quenching, the ζ-potential values of the TrpHA-Au NCs change from +18.5 mV to nearly ~−20 mV, as Figure 5b shows. Most probably, the Cu^2+^ can liberate dissociable protons from the surface-bounded ligand and form complexes on the cluster surface. This is confirmed by the change in the pH of the dispersion from pH = 4.58 to pH = 3.85 (the pH of the pure Cu^2+^ solution is pH = 4.93). Temperature-dependent PL quenching experiments were also carried out to give more information on the nature of the quenching mechanism which is presented in the next section. For Fe^3+^, the LOD value and thus the linear range cannot be determined. Only the smallest detectable amount can be given, which is ~11.0 µM. In this case, the ζ-potential value of the TrpHA-Au NCs does not change measurably, which supports the dominance of other mechanisms for quenching, in contrast to the results obtained for Cu^2+^. The change in the pH of the cluster dispersion was not changed dominantly; only the mixing of the two aqueous solutions (NCs’ dispersion + Fe^3+^ solution) caused a slight pH decrease to pH ~3.2. Furthermore, after the addition of both metal ions, the absorbance spectra do not support the hydrolysis of the metal ions, and the appearance of colored species was not detectable in the visible range which confirms the fact that the stabilizing ligand cannot be eliminated from the metallic surface which was previously determined for histidinehydroxamic acid-stabilized Au NCs [26].

### 3.4. Proposed Mechanism for Interaction of TrpHA-Au NCs with Cu^2+^

To give more information on the quenching mechanism for Cu^2+^ and Fe^3+^, detailed PL quenching studies were conducted at different quencher concentrations and, for Cu^2+^, at different temperatures. As it was previously mentioned, at 25 °C, the fluorescence intensity of TrpHA-Au NCs was shown to quench almost linearly as the concentration of Cu^2+^ increased (Figure 6a,c). On the other hand, in the case of Fe^3+^, the fluorescence intensity was almost constant at low metal concentrations (10.0 µM), and after a sharp decrease above the concentration limit, the extent of quenching became constant again, as can be noticed in Figure 6b. In view of the aforementioned linear relationship, in the case of Cu^2+^, the PL experiments were performed at five different temperatures to recognize the mechanism and thermodynamics of the binding process of Cu^2+^ with TrpHA-Au NCs (Figure 6d). The PL quenching data were evaluated by using the modified Stern–Volmer equation (Equation (2)):

(2) $$log\left[\left(I_{0}/I\right)-1\right]=n\log\left[Q\right]+logK_{b}\;,$$

where the I_0_ and I are the measured intensities before and after the addition of Cu^2+^, K_b_ is the binding constant, n is the number of binding stoichiometry of Cu^2+^ to the TrpHA-Au NCs and [Q] is the applied analytical Cu^2+^ concentration. A similar linear dependence was found in the log [(I_0_/I)−1] vs. log [Q] plots at various temperatures, as represents in Figure 6c, and the slope and intercept of the linear regression were equal to the value of n and log K_b_, respectively. As Table 1 presents, the K_b_ values at three temperature values were in the order of 10^2^ M^−1^. This refers to the fact that there was a dominant interaction, which led to the quenching of the PL intensity due to the appearance of a non-fluorescent adduct.

To have a deeper understanding of the thermodynamics of the reaction between Cu(II) and TrpHA-Au NCs, we evaluated the temperature dependence of the K_b_ values by the following integrated form of the van’t Hoff relation (Equation (3)):

(3) $$\ln K_{b}=\frac{-∆H^{0}\left(T^{0}\right)}{RT}+\frac{∆S^{0}\left(T^{0}\right)}{R}+\frac{∆C_{p}}{R}\left[\left(\frac{T-T^{0}}{T}\right)-ln\frac{T}{T^{0}}\right]$$

This expression was applied to fit the experimentally determined and the estimated values of K_b_ as a function of 1/T, resulting in the values of thermodynamic state functions as parameters of a nonlinear parameter estimation method (Figure 6d). In order to calculate the standard deviations of the thermodynamic parameters, the weighted resampling “jackknife” procedure was applied. Values and standard deviations of enthalpy (ΔH^0^), entropy (ΔS^0^) and heat capacity changes (ΔC_p_) are listed in Table 1. According to the sign of ΔH^0^ and ΔS^0^, there are three potential ways of having an interaction between the interacting species: (1) ΔH^0^ > 0 and ΔS^0^ > 0, hydrophobic forces; (2) ΔH^0^ < 0 and ΔS^0^ < 0, van der Waals forces and hydrogen bonds; and (3) ΔH^0^ < 0 and ΔS^0^ > 0, electrostatic interactions [34]. The negative ΔH^0^ and ΔS^0^ suggest that there were van der Waals forces (e.g., π- π stackings of the aromatic rings) and hydrogen bonds of interaction. DLS studies (Figure S9) confirm a slight aggregation of the NCs by the continuous adding of Cu^2+^, which is further supported the combined quenching process. The complexation property of TrpHA with Cu^2+^ is well-known in the literature [31,32]. In an acidic pH range (pH = 3–5), the formation of [CuL]^+^ and [Cu_2_L_2_H_-1_]^+^ is dominant, while at pH > 4, [CuL_2_] also starts to appear. For the latter two cases, one metal ion can bind with two ligands via the hydroxamate [O,O] and [NH_2_, N_hydroxamate_] or two [NH_2_, N_hydroxamate_], which further confirms the possible connection of the NCs via copper(II)ions, thus allowing the formation of larger aggregates. The above-mentioned van der Waals forces may also arise from the connection of the cluster cores.

### 3.5. Proposed Mechanism for Interaction of TrpHA-Au NCs with Fe^3+^

**Figure 7:**
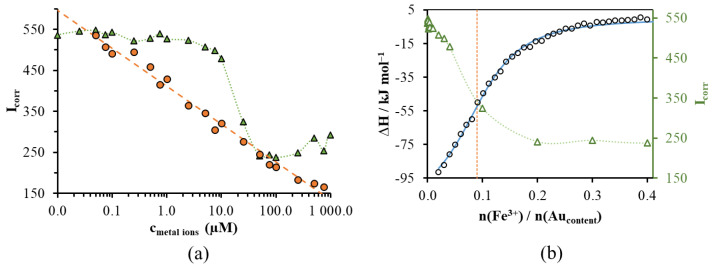
(**a**) Corrected fluorescent intensity of TrpHA-Au NCs in the presence of increasing concentrations of Cu^2+^ (●, orange dots) and Fe^3+^ ions (▲, green triangles) (λ_ex_ = 380 nm, λ_em_ = 470 nm). (**b**) Experimental (○, black circles) and simulated (continuous blue curve) ITC enthalpograms of titration of 1.0 mM Au content TrpHA-Au NCs with 2.0 mM Fe^3+^ ions compared to the corrected fluorescent intensity (Δ) of analogous samples as a function of the metal ion/gold molar ratio.

As opposed to the outcome seen for Cu^2+^, the fluorescence intensity is almost constant at low Fe^3+^ concentrations (Figure 6b and Figure 7a). A significant degree of quenching occurs only above a metal concentration over 10 µM, above which the fluorescence intensity drops sharply and then again assumes a nearly constant value as the concentration of Fe^3+^ reaches 50 µM. These observations allow us to conclude that Fe^3+^ ions form a binding complex with TrpHA-Au NCs with a much higher affinity than Cu^2+^. At 0.50 mM and 1.0 mM metal ion concentration, the same PL aggregation-induced quenching effect can be obtained, which is confirmed by a similar change in the PL lifetime values (Figure S10).

Since there was no possibility for a thermodynamic analysis based on the spectral data, in the case of Fe^3+^, we estimated the degree of thermodynamic quantities accompanying the formation of the bond responsible for the decrease in fluorescence intensity using the isotherm titration calorimetry (ITC) method. Figure 7b shows the ITC results of the binding of Fe^3+^ to the TrpHA-Au NCs, along with the single-binding-site-model-generated theoretical enthalpogram. In addition to the ITC data, Figure 7b also illustrates the change in PL intensity for samples with the same composition as a function of the Fe^3+^/Au molar ratio (this corresponds to the number of atoms). According to the results summarized in Figure 7b, the functions showing a change in the ITC and PL data are altering in an analogously way as a function of the molar ratio. To convert the molar ratios to concentration values, the ITC data confirms that the fluorescence property of the TrpHA-Au NCs does not change significantly below 10 µM of Fe(III) concentration. However, when this limit value is exceeded, Fe^3+^ and TrpHA-Au NCs form a stable adduct (well-defined smaller aggregates) with reduced fluorescence. If the cluster core contains about five gold atoms, the inflection point of the curves is approximately one Fe^3+^-ions per two cluster cores. This calculation may refer to the fact that a metallic core contains one surface-bounded ligand. According to the ITC results, the following parameters can be calculated using the single binding model: K_a_ = 4.67∙10^4^ ± 3.60∙10^3^ M^−1^; ΔG^0^ = −26.64 ± 0.19 kJ∙mol^−1^; ΔH^0^ = −114.14 ± 3.66 kJ∙mol^−1^; and ΔS^0^ = 0.29 kJ∙mol^−1^ K^−1^. Comparing the thermodynamic data, it can be stated that Fe^3+^ has a higher binding affinity to TrpHA-Au NCs than Cu^2+^, but the Cu^2+^ ions can be detected in the presence of Fe^3+^ ions as well, if the Fe^3+^ concentration does not exceed the 10 µM value.

## 4. Conclusions

**Figure 8:**
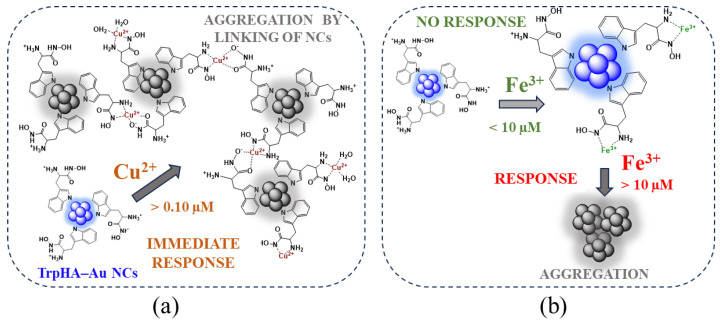
Schematic representation of the proposed mechanisms of the interaction of TrpHA-Au NCs with Cu^2+^ (**a**) and Fe^3+^ (**b**) ions depending on the concentration of the metal ions in acidic (pH = 3–4) conditions.

In this work, the applicability of the hydroxamic acid derivative of *L*-tryptophan was clearly highlighted for the successful fabrication of a few atomic Au NCs with an intense blue emission. For the synthesis, there was no extra use of reducing agents in the aqueous medium. Besides the optical and structural characterization, the dominant role of these Au NCs in sensing application was studied in detail. It was confirmed that the change in the carboxylate group to the hydroxamic acid function dominantly tunes the selectivity as well as the sensitivity. Our sensor system was able to detect only Cu^2+^ with the detection limit of 3.16 µM in the presence of Fe^3+^ (c_Fe(III)_ < 10 µM). Through temperature-dependent fluorescence quenching and quantitative calorimetry studies, it was confirmed that these metal ions can interact with the NCs via different ways, which is summarized by Figure 8a,b.

On one hand, Figure 8 also presents the proposed structure of the TrpHA-stabilized blue-emitting NCs, where the possible binding of the ligand to the NCs’ surface via indole-*N* is demonstrated, but the binding of amino-*N* also presumably occurs, despite FT-IR measurements indicating only the role of the indole ring. By decreasing the sensitivity of our sensor system for Fe^3+^ (LOD ~11 µM), it was demonstrated that the selectivity for Cu^2+^ can be significantly increased (LOD = 3.16 µM). In the case of Cu^2+^, we obtained the result that the TrpHA surface-stabilizing molecules can coordinate Cu^2+^, confirmed by the main thermodynamic parameters which strongly generate the linking of the NCs in acidic pH. The formation of dimer and bis-complexes highly prefers this linkage of the NCs via Cu^2+^ (Figure 8a). In the case of Fe^3+^, the degree of quenching strongly depends on the metal ion concentration, and it only occurs when the NCs are not able to bind more Fe^3+^ on the surface, causing the aggregation of the NCs (Figure 8b). Most probably, via the Fe^3+^ binding, only mono complex(es) are formed in acidic pH (pH ~3.1), but detailed solution equilibrium studies for Fe^3+^-TrpHA are not available in the literature. This different complexation behavior strongly contributes to the sensing process.

## Figures and Tables

**Table 1:** Stern–Volmer binding parameters from linear regression of the log [(I_0_/I)-1] vs. log [Q] representations and thermodynamic parameters from nonlinear regression of the van ‘t Hoff representation of ln K_b_ vs. 1/T data for Cu^2+^ with TrpHA-Au NC interaction at several temperatures.

T (K)	K_b_ (M^−1^)	n	ΔH^0^ (kJ∙mol^−1^)	ΔS^0^ (kJ∙mol^−1^∙K^−1^)	ΔC_p_ (kJ∙mol^−1^∙K^−1^)
283	2.39 × 10^2^ ± 9.09 × 10^−1^	0.5 ± 0.1	−29.69 ± 3.40	−0.11 ± 0.01	−7.19 ± 0.31
293	6.11 × 10^1^ ± 8.72 × 10^−1^	0.4 ± 0.1			
303	4.56 × 10^1^ ± 8.72 × 10^−1^	0.3 ± 0.1			
313	1.24 × 10^2^ ± 8.72 × 10^−1^	0.4 ± 0.1			
318	3.34 × 10^2^ ± 8.99 × 10^−1^	0.5 ± 0.1			

## Data Availability

Data are available on request.

## Supplementary Materials

The following supporting information can be downloaded at: https://www.mdpi.com/article/10.3390/nano14050434/s1. Figure S1: Synthesis protocol of the TrpHA ligand; Figure S2: ^1^H-NMR spectrum of TrpHA; Figure S3: ESI-MS spectrum of TrpHA; Figure S4. Representative emission spectra of TrpHA-Au NCs-containing dispersion using different TrpHA concentration (c_Au_ = 1.0 mM, pH = 7.0); Figure S5: Emission spectra of the TrpHA-Au NCs after different treatments (c_Au_ = 1.0 mM (after 48 h synthesis time); Figure S6: Absorbance spectra of the HAuCl_4_ solution (c = 0.1 mM), the pure TrpHA ligand (c = 1.0 mM) and the TrpHA-Au NCs (c_Au_ = 0.95 mM); Figure S7. FT-IR spectra of the lyophilized powder form of the TrpHA and TrpHA-Au NCs in the range of 4000–2400 cm^−1^ (a) and 1800–1000 cm^−1^ (b) under same pH; Figure S8. Representative HRTEM images of TrpHA-Au NCs-containing aqueous dispersion at different enlargements; Figure S9: DLS curves of the TrpHA-Au NCs in the absence and in the presence of increasing concentration of Cu^2+^ ions; Figure S10: The typical fluorescence decay curves of the TrpHA-Au NCs before (black) and after the addition of Cu^2+^ (blue) and Fe^3+^ (green) ions using 1 mM metal ion concentration (c_Au_ = 0.25 mM).
