# Supplementary material for: Tryptophanhydroxamic Acid-Stabilized Ultrasmall Gold Nanoclusters: Tuning the Selectivity for Metal Ion Sensing
Source: Nanomaterials (Basel). 2024 Feb 27;14(5):434. doi: 10.3390/nano14050434 (PMC10933814; doi:10.3390/nano14050434)
Supplement: Supplementary file 1 [file nanomaterials-14-00434-s001.zip › nanomaterials-2874710-supplementary.pdf]

SUPPORTING INFORMATION

Tryptophanhydroxamic Acid-Stabilized Ultrasmall Gold Nanoclusters: Tuning the Selectivity for Metal Ion Sensing

Gyöngyi Gombár [1], Ditta Ungor [1], István Szatmári [2,3], Ádám Juhász [1,4,*] and Edit Csapó [1,4,*]

[1] MTA-SZTE Lendület "Momentum" Noble Metal Nanostructures Research Group, University of Szeged, Rerrich B. sqr. 1, H-6720 Szeged, Hungary
[2] Institute of Pharmaceutical Chemistry, University of Szeged, Eötvös u. 6, H-6720 Szeged, Hungary
[3] Stereochemistry Research Group, Eötvös Loránd Research Network, University of Szeged, Eötvös u. 6, H-6720 Szeged, Hungary
[4] Interdisciplinary Excellence Center, Department of Physical Chemistry and Materials Science, University of Szeged, Rerrich B. sqr. 1, H-6720 Szeged, Hungary
* Correspondence: juhaszad@chem.u-szeged.hu (Á.J.); juhaszne.csapo.edit@med.u-szeged.hu (E.C.)

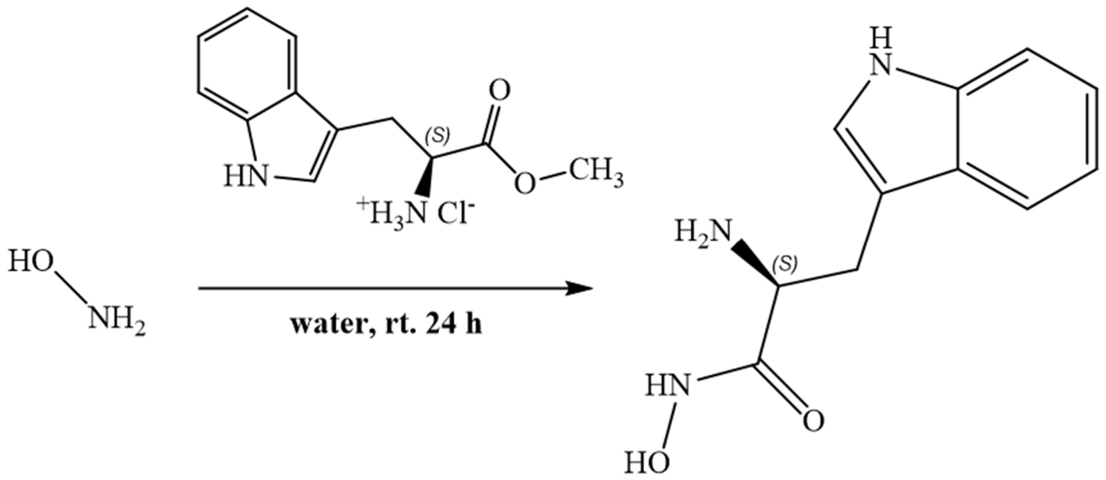

Figure S1. Synthesis protocol of the TrpHA ligand.

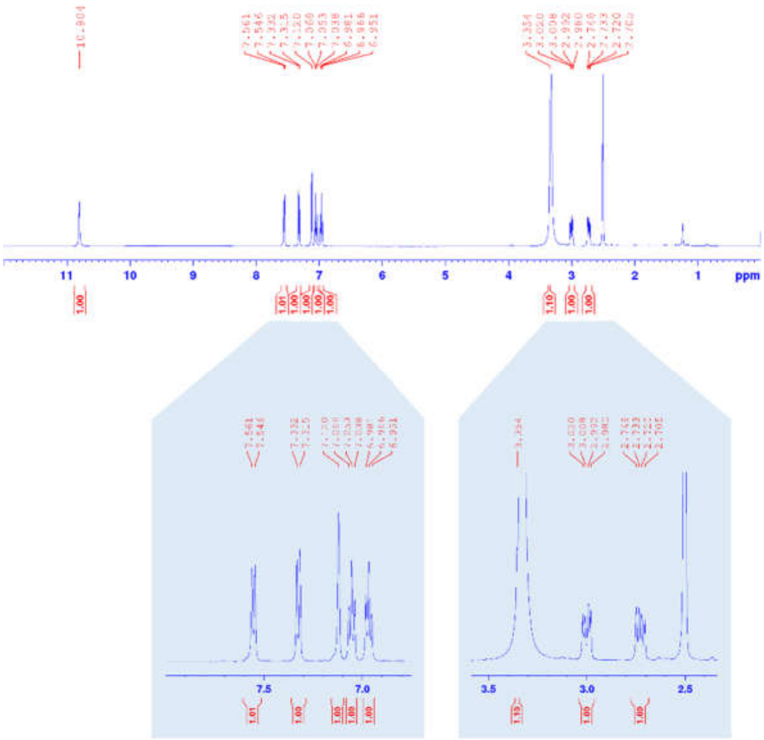

Figure S2. ^{1}H-NMR spectrum of TrpHA.

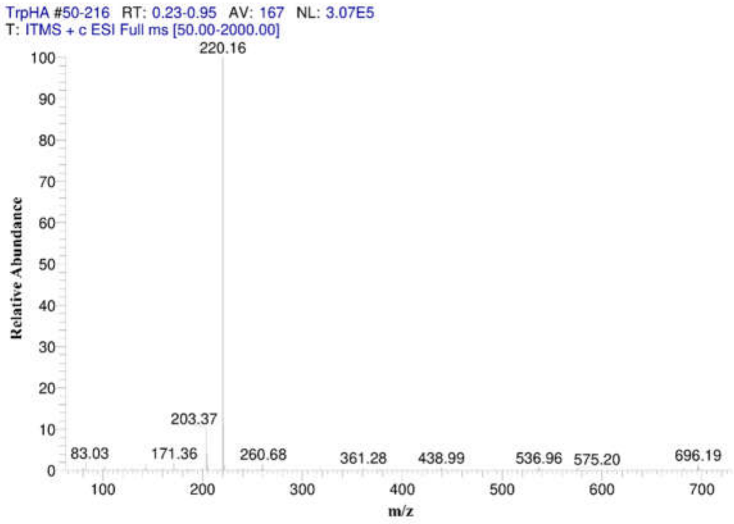

Figure S3. ESI-MS spectrum of TrpHA.

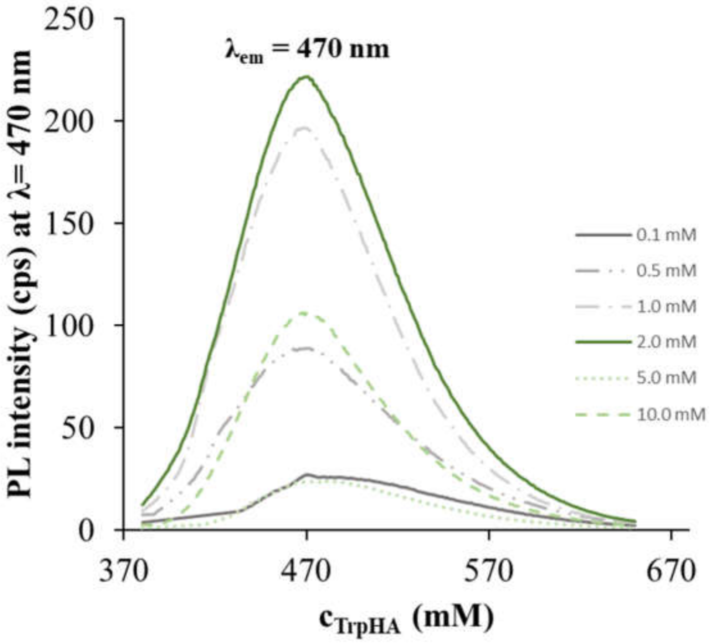

Figure S4. Representative emission spectra of TrpHA-Au NCs-containing dispersions using different TrpHA concentration (c_{Au} = 1.0 mM, pH = 7.0).

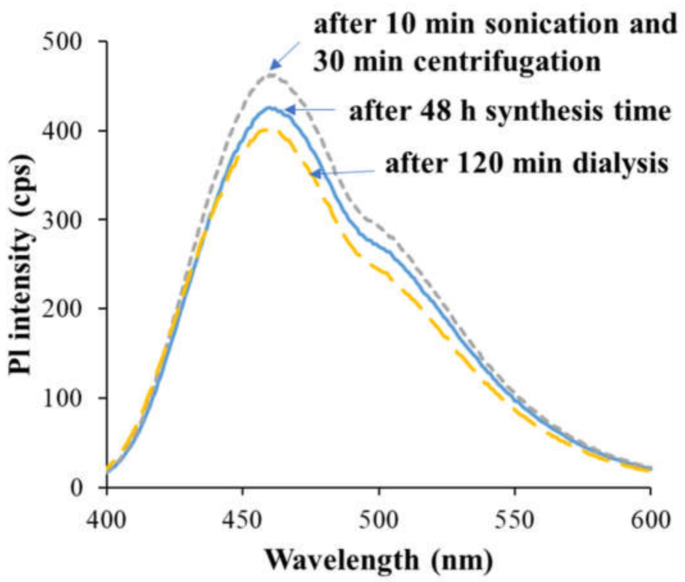

Figure S5. Emission spectra of the TrpHA-Au NCs after different treatments (c_{Au} = 1.0 mM, after 48 h synthesis time)

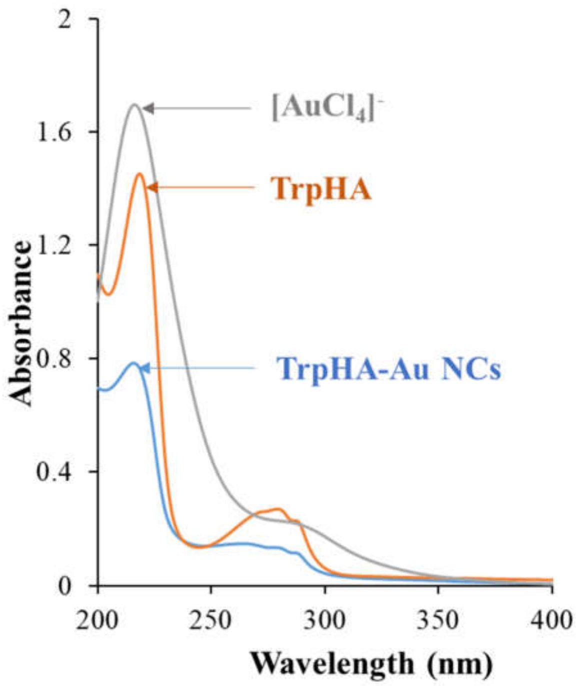

Figure S6. Absorbance spectra of the $[AuCl_4]^-$ (c = 0.1 mM), the pure TrpHA ligand (c = 1.0 mM) and the TrpHA-Au NCs (c_{Au} = 1.0 mM).

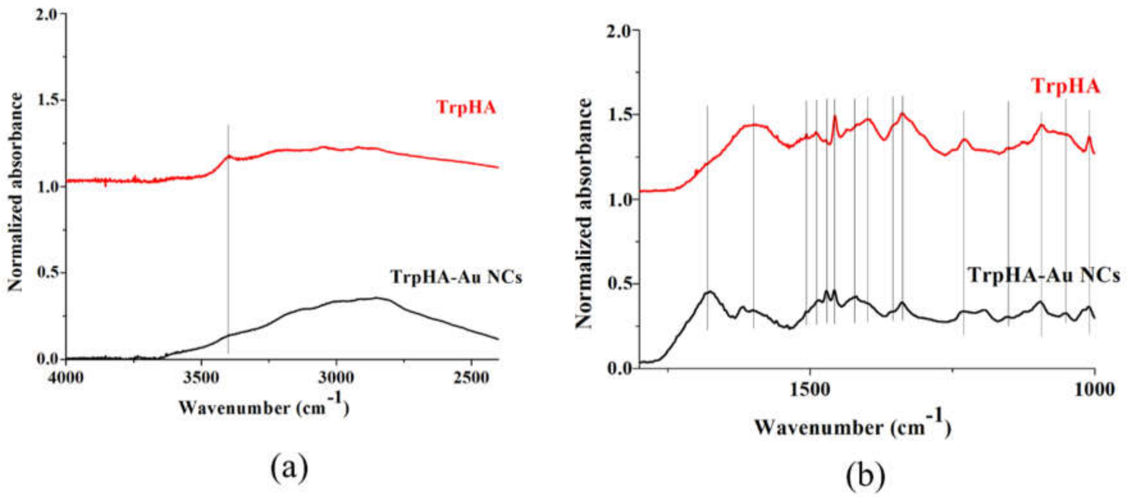

Figure S7. FT-IR spectra of the lyophilized powder form of the TrpHA and TrpHA-Au NCs in the range of 4000-2400 cm^{-1} (a) and 1800-1000 cm^{-1} (b) under same pH.

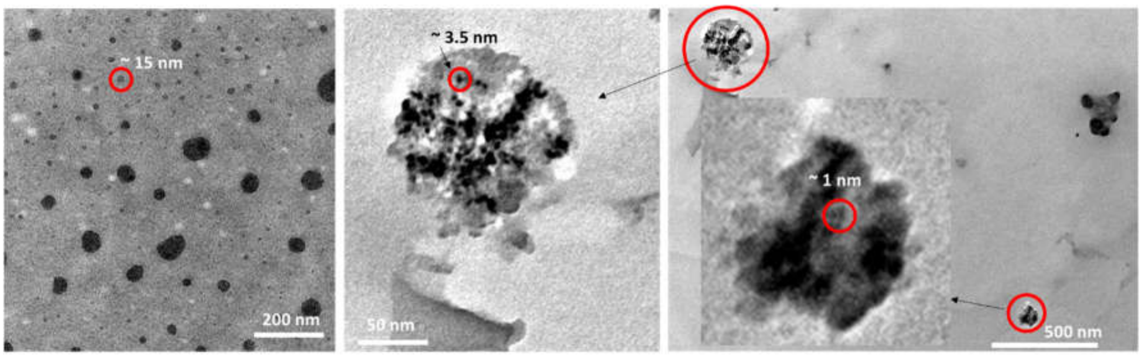

Figure S8. Representative HRTEM images of TrpHA-Au NCs-containing aqueous dispersion at different enlargements.

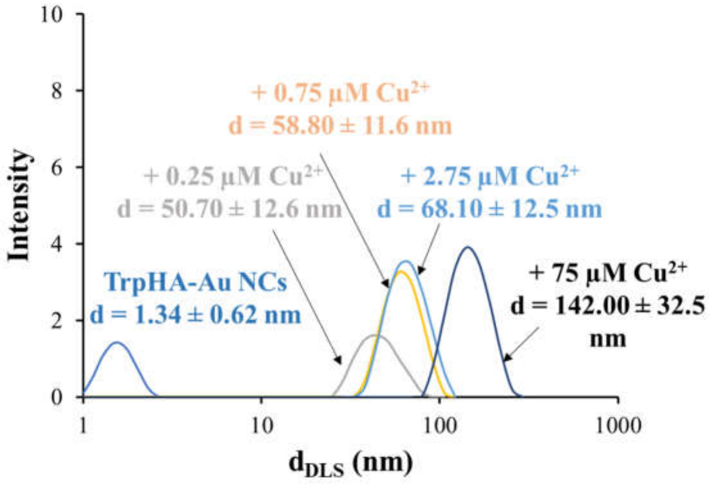

Figure S9. DLS curves of the TrpHA-Au NCs in the absence and in the presence of increasing concentration of Cu^{2+}-ions

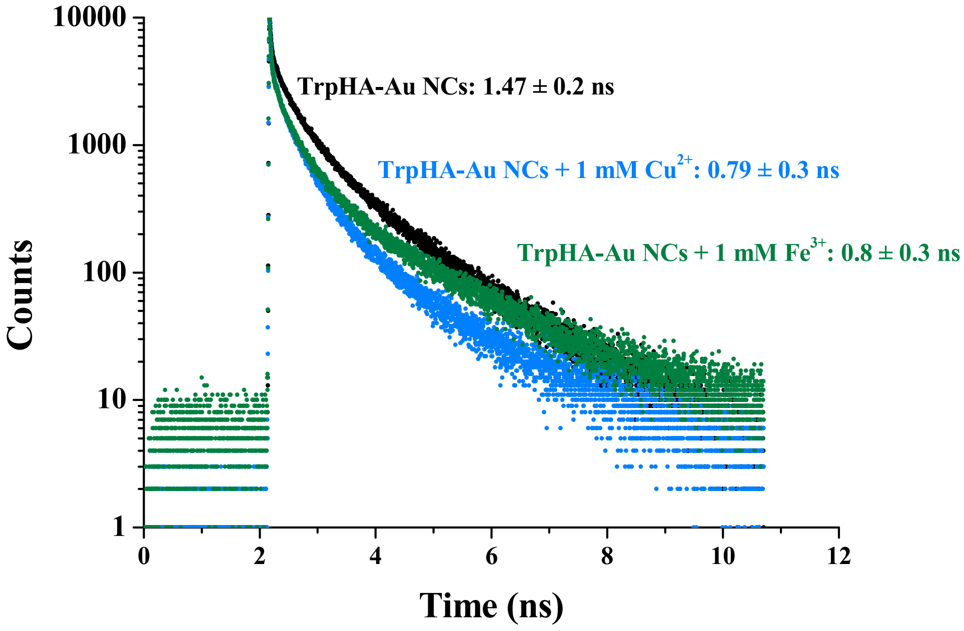

Figure S10. The typical fluorescence decay curves of the TrpHA-Au NCs before (black) and after the addition of Cu^{2+}- (blue) and Fe^{3+} - (green) ions using 1 mM metal ion concentration (c_{Au} = 0.25 mM)
